# Data on social transmission of food preference in a model of autism induced by valproic acid and translational analysis of circulating microRNA

**DOI:** 10.1016/j.dib.2018.04.047

**Published:** 2018-04-21

**Authors:** Mauro Mozael Hirsch, Iohanna Deckmann, Mellanie Fontes-Dutra, Guilherme Bauer-Negrini, Gustavo Della-Flora Nunes, Walquiria Nunes, Bruna Rabelo, Rudimar Riesgo, Rogerio Margis, Victorio Bambini-Junior, Carmem Gottfried

**Affiliations:** a Translational Group in Autism Spectrum Disorder - GETTEA, Clinical Hospital of Porto Alegre, RS, Brazil; b Department of Biochemistry, Federal University of Rio Grande do Sul - UFRGS, Porto Alegre, RS, Brazil; c Brazilian National Institute of Science and Technology on Neuroimmunomodulation (INCT-NIM), Rio de Janeiro, Brazil; d Child Neurology Unit, Clinical Hospital of Porto Alegre, Federal University of Rio Grande do Sul, Porto Alegre, Brazil; e Center of Biotechnology and PPGBCM, Laboratory of Genomes and Plant Populations, Federal University of Rio Grande do Sul - UFRGS, Porto Alegre, RS, Brazil; f School of Pharmacy and Biomedical Sciences, University of Central Lancashire, Preston, UK

**Keywords:** Autism, Social transmission of food preference, microRNA, Resveratrol, Translational research, Preclinical models, Valproate

## Abstract

This article contains data of Social Transmission of Food Preference in an animal model of autism and the evaluation of a set of microRNA analyzed in autistic patients and animal model of autism. The analyses of the absolute consumption of two flavored food by male rats prenatally exposed to valproic acid (VPA) and treated with resveratrol (RSV), showed that VPA animals show a trend to eat less of the flavored food presented by a demonstrator rat. We also identified 13 microRNA with similar levels among rodents’ experimental groups, as well as 11 microRNA with no alterations between autistic and control subjects. Further evaluation of mechanisms of VPA and RSV actions on behavioral and molecular alterations can shed light in important biomarkers and etiological triggers of autistic spectrum disorders.

**Specifications Table**

**Table:** 

Subject area	*Pharmacology, Toxicology and Pharmaceutical Science*
More specific subject area	*Autism Spectrum Disorder*
	*Toxicology*
	*Natural products*
Type of data	*Table, Figures*
How data was acquired	*Animal behavior analysis and Reverse Transcription followed by Quantitative Polymerase Chain Reaction (RT-qPCR)*
Data format	*Analyzed*
Experimental factors	*The animal model of autism was induced by a single intraperitoneal injection of 600 mg/kg VPA or saline solution on embryonic day 12.5 (E12.5). RSV treatments were achieved by daily subcutaneous injections of RSV (3.6 mg/kg) or DMSO from E6.5 to E18.5. Blood samples from these animals were obtained by cardiac puncture 30 days after birth. Peripheral blood samples from autistic male individuals and from the control group (5–15 years-old range) were obtained at Clinical Hospital of Porto Alegre (HCPA).*
Experimental features	*Social Transmission of Food Preference (STFP) test was performed in male rats after food habituation, consumption of one of flavored food by demonstrator rat and interaction between demonstrator and observer rats. The amount of cued and non-cued food eaten by observers from each litter was weighed and recorded. After homogenization of blood samples, we performed RNA extraction and the mature miRNA expression was evaluated by reverse transcription followed by quantitative polymerase chain reaction (RT-qPCR), using fluorescence of SYBR Green to detect amplification, estimate Ct values and to determine specificity after melting curve analysis.*
Data source location	*Department of Biochemistry, Federal University of Rio Grande do Sul - UFRGS, Porto Alegre, RS, Brazil. Clinical Hospital of Porto Alegre, Porto Alegre, RS, Brazil.*
Data accessibility	*Hirsch, M. M. et al. Behavioral alterations in autism model induced by valproic acid and translational analysis of circulating microRNA.* Food Chem. Toxicology 115 (2018): 336–343. doi: 10.1016/j.fct.2018.02.061.[1]

**Value of the data**

• Social transmission by absolute consumption of two flavored food in VPA-induced animal model of autism.
• MicroRNA analysis in blood samples from male rats prenatally exposed to VPA and treated with RSV, compared to control animals.
• MicroRNA analysis in blood samples from 5 to 15 years old autistic subjects.

## 1 Data

**Fig. 1:**
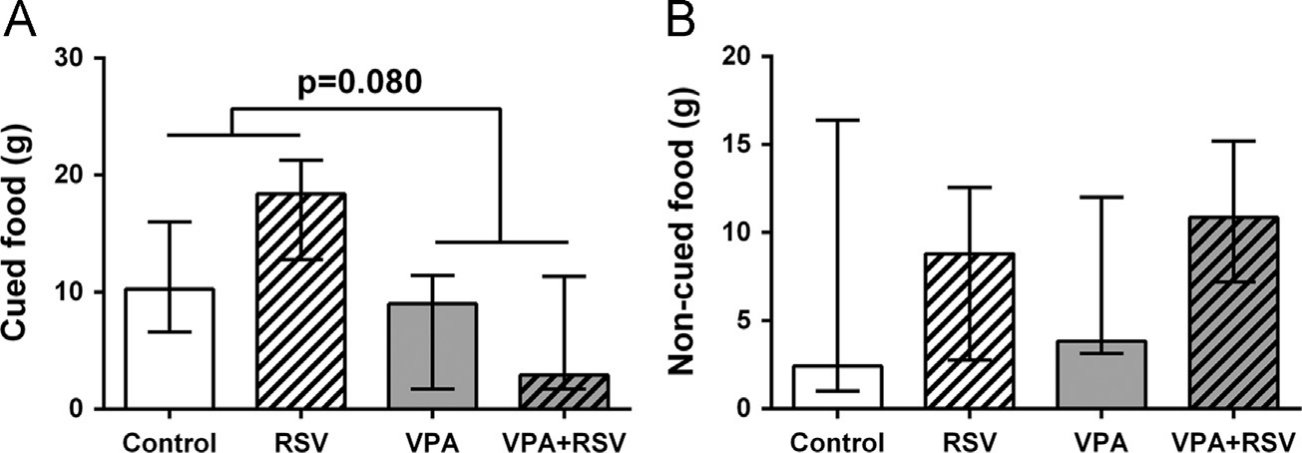
Absolute weights of consumed food in VPA autism model. Cued (A) and non-cued (B) food. Plots show medians±interquartile intervals. Statistical analysis: Independent-Samples Kruskal–Wallis test. Control (*n*=8), RSV (*n*=9), VPA (*n*=9), VPA+RSV (*n*=6).

Fig. 1 presents the absolute food consumption of Wistar rats prenatally exposed to VPA, compared to the control animals, after treatment with RSV or vehicle. The animals exposed to VPA have a tendency to eat less of the flavored-food cued by demonstrator (Fig. 1A, *p*=0.080). On the other hand, animals presented no differences in consumption of alternative (non-cued) food across interventions (Fig. 1B, *p*=0.493).

**Fig. 2:**
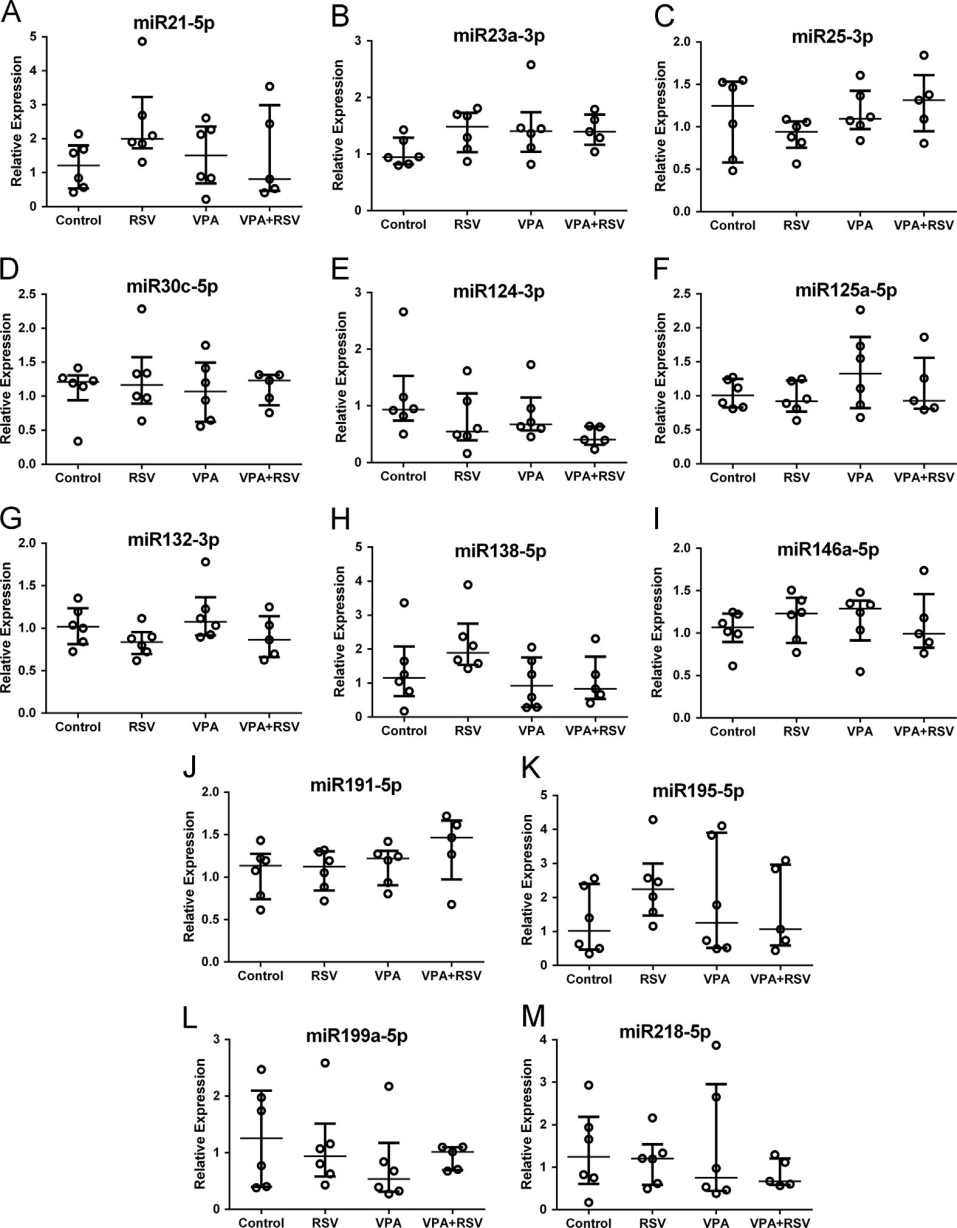
Relative expressions of unchanged miRNA in peripheral blood of VPA autism model at P30. Plots presented as medians±interquartile intervals. Statistical analysis: Independent-Samples Kruskal–Wallis test. Control (*n*=6), RSV (*n*=6), VPA (*n*=6), VPA+RSV (*n*=5).

We evaluated the relative expression of miRNA in blood of 30 days rats prenatally exposed to VPA or vehicle (saline) and treated with RSV or vehicle (DMSO). The GeNorm algorithm ranked miRNA miR181a-5p and miR181b-5p as the most stable ones and they were used as normalizers to evaluate the relative expression of the remaining miRNA. We observed no significant differences in levels of 13 miRNA (Fig. 2).

**Fig. 3:**
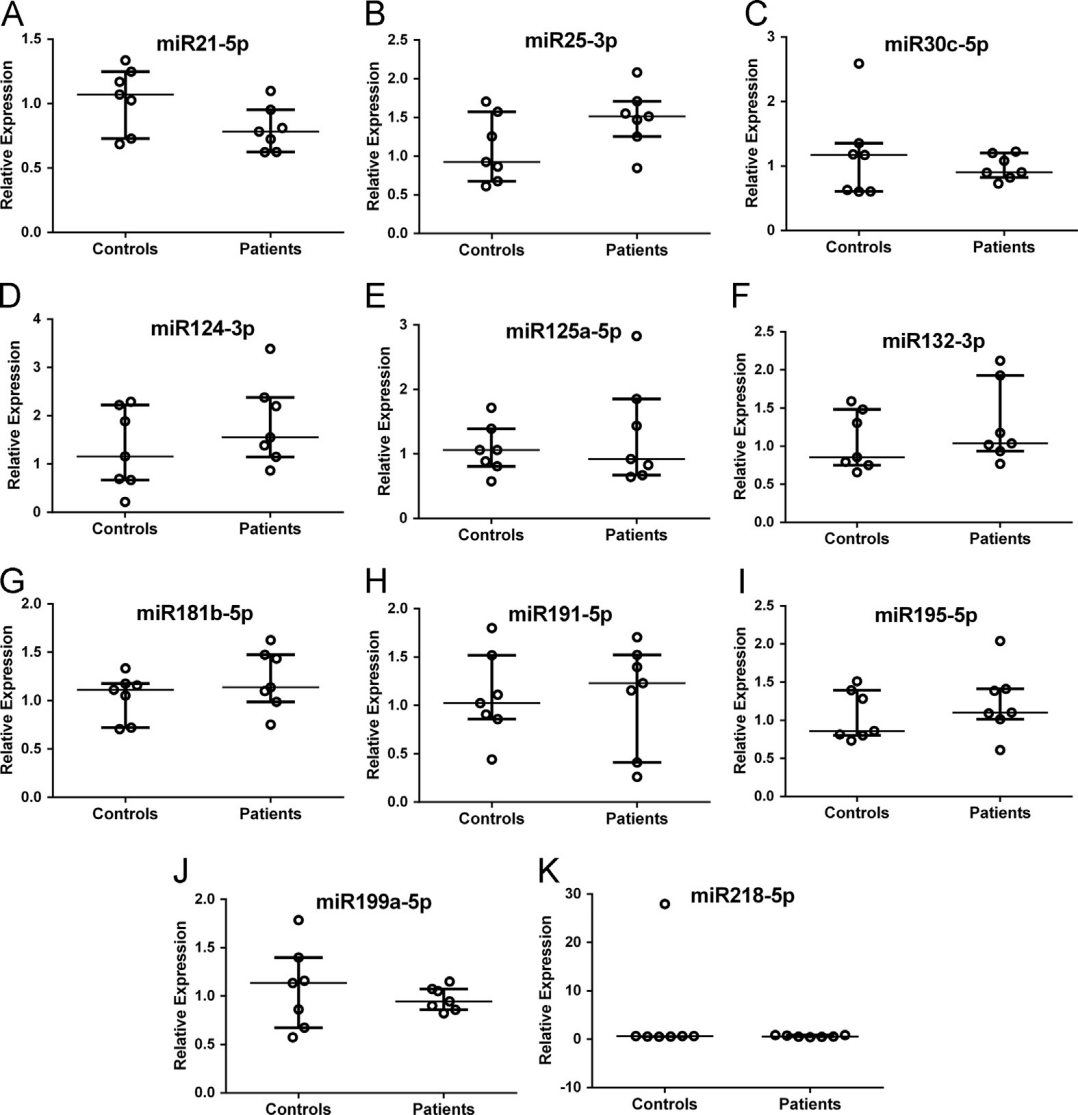
Relative expressions of uncharged miRNA in peripheral blood of autistic children compared to control group. Results expressed as medians±interquartile intervals. Statistical analysis: Mann–Whitney U-test. Control (*n*=7) and autistic patients (*n*=7).

We also determined the relative expression of a set of miRNA in peripheral blood of autistic subjects, compared with controls in the same age-range (5–15 years). The GeNorm algorithm ranked miR23a-3p, miR146a-5p and miR181a-5p as the most stable ones and they were used as normalizers to evaluate the relative expression of the remaining miRNA. There were no intra-group differences related to age on miRNA levels. The analysis of relative expression revealed no significant differences in 11 miRNA in peripheral blood of autistic children, compared to control subjects (Fig. 3).

## 2 Experimental design, materials and methods

### 2.1 Animal model of autism induced by VPA and resveratrol treatment

The animal model of autism was induced as previously described [2], [3]. Briefly, female Wistar rats (UFRGS-Biochemistry Department CREAL), with controlled fertility cycles, were mated overnight. The first day of gestation was determined by the presence of spermatozoa in the vaginal smear (embryonic day 0.5). Pregnant females received a single intraperitoneal injection of 600 mg/kg VPA (sodium valproate, Sigma-Aldrich, USA) or saline solution on embryonic day 12.5 (E12.5), and daily subcutaneous injections of RSV (trans-resveratrol, Fluxome, Stenløse, Denmark, 3.6 mg/kg) or DMSO from E6.5 to E18.5 [4]. Only males of the offspring were used in this study. Blood samples from these animals were obtained by cardiac puncture 30 days after birth. This project was approved by the local animal ethics committee (CEUA-UFRGS 140367/140431) and all animals were handled in accordance with established guidelines (National Council for the Control of Animal Experimentation (CONCEA)).

### 2.2 Evaluation of food consume in *STFP test*

This experiment was adapted from Wrenn et al. protocol [5]. Rats at 47 days of age were habituated for 72 h to eat pelleted food made from powdered chow. In the following day, food was removed three hours before the test. Next, one animal from each cage (demonstrator) was housed alone in a separate housing box and was allowed to eat a randomly assigned flavored food for 1 h: either cinnamon (1% w/w) or cocoa (2% w/w). Then the demonstrator rat was housed with their littermates (the observer rats) and allowed free interaction for 30 min. After this interaction period, the demonstrator animal was removed from the housing box and the observer animals were provided with two choices of powdered food in identical pellets, one with flavor of the cued food presented by demonstrator rat and the other with the alternative food. Rats were allowed to eat for one and a half hours, and the amount of cued and non-cued food eaten from each litter was weighed and recorded.

### 2.3 Blood samples from autistic and control subjects

Peripheral blood samples from autistic male individuals and from the control group (5–15 years-old range) were obtained at the Clinical Hospital of Porto Alegre (HCPA). Inclusion criteria were age 5–15 years, have a clinical diagnosis of ASD according to DSM-5 and confirmed using the Autism Diagnostic Observation Schedule. Autistic individuals who presented secondary autism or autism as an associated feature to an identified genetic condition (Fragile X Syndrome, Rett Syndrome, Angelman Syndrome, Prader-Willi Syndrome, Smith-Lemli-Opitz Syndrome and Tuberous Sclerosis) were excluded from the study. This project was approved by the local ethics committee (CEUA-UFRGS 33863).

### 2.4 RNA extraction and RT-qPCR procedure

After homogenization of blood samples with Trizol^®^ reagent (Invitrogen, USA), chloroform was added to perform phase separation, and RNA was precipitated from the upper aqueous layer using isopropanol. The precipitated RNA was washed with ethanol to remove impurities, resuspended in RNase-free water and stored at −80 °C [6].

Mature miRNA expression was evaluated by reverse transcriptase followed by quantitative polymerase chain reaction (RT-qPCR) [7]. Complementary DNA (cDNA) was synthesized from mature miRNA using reverse transcriptase reaction containing 2 µg of total RNA, 1 µL of 10 mM dNTP mix (Invitrogen, USA), 3 µL of stem loop RT primer mix (Supplementary Table 1), 4 µL M-MLV reverse transcriptase 5× reaction buffer (Invitrogen, USA), 2 µL of 0.1 M DTT (Invitrogen, USA), 1 µL of RNase inhibitor (Invitrogen, USA), 1.0 µL of M-MLV reverse transcriptase (Invitrogen, USA), and sterile distilled water to a final volume of 20 µL. The synthesis of the cDNA was completed after a sequence of three incubations at 65 °C for 5 min, 37 °C for 50 min and 70 °C for 15 min.

The quantitative PCR mix was comprised by 12 µL of cDNA (1:33), 1.0 µL of specific miRNA forward and universal reverse (10 µM) primers (as detailed in Table 1), 0.5 µL of 10 µM dNTP mix, 2.4 µL of 10× PCR buffer (Invitrogen, USA), 0.8 µL of 50 mM MgCl_2_ (Invitrogen, USA), 2.4 µL of 1× SYBR^™^ Green (Molecular Probes, USA), 0.1 µL of Platinum Taq DNA Polymerase (Invitrogen, USA) and sterile distilled water to a final volume of 24 µL. The fluorescence of SYBR^™^ Green was used to detect amplification, estimate Ct values, and to determine specificity after melting curve analysis. PCR cycling conditions were standardized to 95 °C for 5 min followed by 40 cycles at 95 °C for 10 s, 60 °C for 10 s, and 72 °C for 10 s. After the main amplification, sample fluorescence was measured from 60 °C to 95 °C, with an increasing ramp of 0.3 °C each, to obtain the denaturing curve of the amplified products and Tm estimation, to assure their homogeneity after peak detection. Data was obtained from an Applied Biosystems StepOne System (USA). The set of 16 miRNA selected for this study includes miRNA involved in immunological and/or synaptic plasticity modulation, both processes altered in ASD and includes miRNA commonly altered in many neurodevelopmental disorders.

**Table 1:** Primer sequences for 16 miRNA evaluated. Forward and RT stem-loop primers and an universal reverse primer.

**miRNA ID**	**miRNA sequence**	**Primer type**	**Primer sequences**
miR132-3p	uaacagucuacagccauggucg	Forward	TCC GGC TAA CAG TCT ACA GCC A
		RT stem-loop	GTC GTA TCC AGT GCA GGG TCC GAG GTA TTC GCA CTG GAT ACG AC cgacca
miR138-5p	agcugguguugugaaucaggccg	Forward	TCC GGA AGC TGG TGT TGT GAA TC
		RT stem-loop	GTC GTA TCC AGT GCA GGG TCC GAG GTA TTC GCA CTG GAT ACG AC cggcct
miR125a-5p	ucccugagacccuuuaaccuguga	Forward	GTC GCG ATC CCT GAG ACC CTT TA
		RT stem-loop	GTC GTA TCC AGT GCA GGG TCC GAG GTA TTC GCA CTG GAT ACG AC tcacag
miR195-5p	uagcagcacagaaauauuggc	Forward	GGG CGC TAG CAG CAC AGA AAT A
		RT stem-loop	GTC GTA TCC AGT GCA GGG TCC GAG GTA TTC GCA CTG GAT ACG AC gccaat
miR199a-5p	cccaguguucagacuaccuguuc	Forward	GAT GCG CCC AGT GTT CAG ACT
		RT stem-loop	GTC GTA TCC AGT GCA GGG TCC GAG GTA TTC GCA CTG GAT ACG AC gaacag
miR134-5p	ugugacugguugaccagagggg	Forward	GGC TCT TGT GAC TGG TTG ACC A
		RT stem-loop	GTC GTA TCC AGT GCA GGG TCC GAG GTA TTC GCA CTG GAT ACG AC cccctc
miR124-3p	uaaggcacgcggugaaugcc	Forward	CTA GCT TAA GGC ACG CGG TGA
		RT stem-loop	GTC GTA TCC AGT GCA GGG TCC GAG GTA TTC GCA CTG GAT ACG AC ggcatt
miR181a-5p	aacauucaacgcugucggugagu	Forward	GCG CTG AAC ATT CAA CGC TGT C
		RT stem-loop	GTC GTA TCC AGT GCA GGG TCC GAG GTA TTC GCA CTG GAT ACG AC actcac
miR181b-5p	aacauucauugcugucggugggu	Forward	GCT GCG CAA CAT TCA TTG CTG TC
		RT stem-loop	GTC GTA TCC AGT GCA GGG TCC GAG GTA TTC GCA CTG GAT ACG AC acccac
miR25-3p	cauugcacuugucucggucuga	Forward	TCA GCA CAT TGC ACT TGT CTC GG
		RT stem-loop	GTC GTA TCC AGT GCA GGG TCC GAG GTA TTC GCA CTG GAT ACG AC tcagac
miR21-5p	uagcuuaucagacugauguuga	Forward	CCG GCG CTA GCT TAT CAG ACT GAT
		RT stem-loop	GTC GTA TCC AGT GCA GGG TCC GAG GTA TTC GCA CTG GAT ACG AC tcaaca
miR23a-3p	aucacauugccagggauuucc	Forward	GCT GTC ATC ACA TTG CCA GGG A
		RT stem-loop	GTC GTA TCC AGT GCA GGG TCC GAG GTA TTC GCA CTG GAT ACG AC ggaaat
miR146a-5p	ugagaacugaauuccauggguu	Forward	CGT GGC GTG AGA ACT GAA TTC CA
		RT stem-loop	GTC GTA TCC AGT GCA GGG TCC GAG GTA TTC GCA CTG GAT ACG AC aaccca
miR218-5p	uugugcuugaucuaaccaugu	Forward	GCC GTC CTT GTG CTT GAT CTA ACC
		RT stem-loop	GTC GTA TCC AGT GCA GGG TCC GAG GTA TTC GCA CTG GAT ACG AC acatgg
miR30c-5p	uguaaacauccuacacucucagc	Forward	GCG TCG CTG TAA ACA TCC TAC ACT C
		RT stem-loop	GTC GTA TCC AGT GCA GGG TCC GAG GTA TTC GCA CTG GAT ACG AC gctgag
miR191-5p	caacggaaucccaaaagcagcug	Forward	GGA GCG TCA ACG GAA TCC CAA AAG
		RT stem-loop	GTC GTA TCC AGT GCA GGG TCC GAG GTA TTC GCA CTG GAT ACG AC cagctg
Reverse Universal Primer		Reverse	CCA GTG CAG GGT CCG AGG TA

### 2.5 Calculation of miRNA relative expression

The RT-qPCR results were imported into Microsoft Excel and the geNorm program was used to assess the variance in expression levels of the miRNA analyzed [8], [9]. This program scanned the present miRNA two at a time. Then, the expression stabilities of the set of miRNA were evaluated. All miRNA were ranked accordingly to their stability. A pairwise variation analysis was performed by geNorm to determine the number of miRNA required for accurate normalization and to identify the most suitable miRNA to be used as normalizers.

The average value of crossing threshold (Ct) values (in triplicate) was converted to quantities for geNorm analysis by the difference between Ct values from two groups taken in each comparison. PCR efficiency was calculated from the slope of the amplification curve by exponential amplification analysis using the LinRegPCR algorithm [10]. The relative expression was obtained using the −ΔΔCt method where Ct values of a group are subtracted from the average Ct values of the control group. The relative expression of miRNA was calculated considering the PCR efficiency and the –ΔΔCt values for each miRNA [11] and were normalized to the normalizers identified by the geNorm software.

### 2.6 Statistical analysis

IBM SPSS Statistics 20.0 (IBM SPSS, Armonk, NY, USA) was used to perform the statistical analysis. Kolgomorov–Smirnov and Shapiro–Wilk tests of normality were applied to determine data distribution. The absolute consumption of each food flavor presented non-normal distribution, so that were compared by non-parametric Kruskal–Wallis test and the results expressed as median±interquartile interval. The miRNA relative expressions of the four animal groups were compared using non-parametric Kruskal–Wallis test and the results were expressed as median±interquartile interval. For the analysis of the human samples, non-parametric Mann–Whitney U-test was performed and the results were expressed as median±interquartile interval. All statistical analyses were supervised by the Biostatistics Unit at the Clinical Hospital of Porto Alegre.

## References

[1] Hirsch M.M., Deckmann I., Fontes-Dutra M., Bauer-Negrini G., Della-Flora Nunes G., Nunes W., Rabelo B., Riesgo R., Margis R., Bambini-Junior V., Gottfried C. (2018). Behavioral alterations in autism model induced by valproic acid and translational analysis of circulating microRNA. Food Chem. Toxicol..

[2] Bambini-Junior V., Rodrigues L., Behr G.A., Moreira J.C.F., Riesgo R., Gottfried C. (2011). Animal model of autism induced by prenatal exposure to valproate: behavioral changes and liver parameters. Brain Res..

[3] Schneider T., Przewłocki R. (2005). Behavioral alterations in rats prenatally exposed to valproic acid: animal model of autism. Neuropsychopharmacology.

[4] Bambini-Junior V., Zanatta G., Della Flora Nunes G., Mueller de Melo G., Michels M., Fontes-Dutra M., Nogueira Freire V., Riesgo R., Gottfried C. (2014). Resveratrol prevents social deficits in animal model of autism induced by valproic acid. Neurosci. Lett..

[5] Wrenn C.C., Wrenn C.C. (2004). Social transmission of food preference in mice. Curr. Protoc. Neurosci..

[6] Chomczynski P. (1993). A reagent for the single-step simultaneous isolation of RNA, DNA and proteins from cell and tissue samples.. Biotechniques.

[7] Chen C., Ridzon D.A., Broomer A.J., Zhou Z., Lee D.H., Nguyen J.T., Barbisin M., Xu N.L., Mahuvakar V.R., Andersen M.R., Lao K.Q., Livak K.J., Guegler K.J. (2005). Real-time quantification of microRNAs by stem-loop RT-PCR. Nucleic Acids Res..

[8] Peltier H.J., Latham G.J. (2008). Normalization of microRNA expression levels in quantitative RT-PCR assays: identification of suitable reference RNA targets in normal and cancerous human solid tissues. RNA.

[9] Vandesompele J., De Preter K., Pattyn F., Poppe B., Van Roy N., De Paepe A., Speleman F. (2002). Accurate normalization of real-time quantitative RT-PCR data by geometric averaging of multiple internal control genes. Genome Biol..

[10] Ramakers C., Ruijter J.M., Deprez R.H.L., Moorman A.F.M. (2003). Assumption-free analysis of quantitative real-time polymerase chain reaction (PCR) data. Neurosci. Lett..

[11] Pfaffl M.W. (2001). A new mathematical model for relative quantification in real-time RT-PCR. Nucleic Acids Res..

